# Overview of Orphan Medicines in European Union: An Analysis of Regulatory and Technical–Scientific Aspects

**DOI:** 10.1007/s43441-025-00810-1

**Published:** 2025-05-29

**Authors:** Greta Santi Laurini, Victoria Nikitina, Massimiliano Broccoli, Nicola Montanaro, Domenico Motola

**Affiliations:** 1 Unit of Pharmacology, Department of Medical and Surgical Sciences, University of Bologna, Via Irnerio 48, Bologna, 40126 Italy; 2 Alma Mater Studiorum University di Bologna, Bologna, Italy

**Keywords:** Orphan Drugs, Rare Disease, Orphan Designation, European Medicines Agency, Clinical Pharmacology

## Abstract

**Introduction:**

The Orphan Medicinal Product Regulation was adopted in the EU in 2000 to encourage the implementation of medicines for rare diseases. Providing a current overview of its effects, this study was performed on medicines with active orphan designation authorised in the EU until January 17, 2024.

**Materials and methods:**

Based on the Community Register of orphan medicinal products for human use, active orphan designations of medicines that have been granted marketing authorisation (MA) were included in the study. General information on medicines, orphan designations, and MAs was collected from web-based sources and analysed using descriptive statistics.

**Results:**

Since 2000, 149 medicines with clinical indications with active orphan designation have been granted MA in the EU, making a total of 184 authorised orphan indications. Most medicines (96;64.4%) received standard MA, while 33 (22.1%) received conditional MA and 20 (13.4%) MA under exceptional circumstances. Sixty-five (43.6%) medicines were biological products, mainly monoclonal antibodies, recombinant human peptides or enzymes, or gene therapies. Active orphan designations with outcome for MA were primarily for indications for neoplasms or endocrine, nutritional or metabolic diseases. Orphan indications were licensed after a mean of 67.2 months (range 6–249 months) from designation date. For 93 (50.5%) orphan designations, the prevalence estimate of the condition in the EU was ≤ 1/10,000.

**Conclusions:**

Despite pharmacological advances, a limited number of orphan medicines have been authorised in the EU since the entry into force of the Orphan Regulation, making the lack of available medicines for rare diseases still a public health problem.

## Introduction

Despite pharmacological advances, rare diseases are still a major public health problem affecting about 30 million people within the European Union (EU) [1]. Targeting rare diseases, so-called orphan medicines face specific challenges for development and marketing resulting in little commercial interest from the pharmaceutical industry in this field. Among the issues raised by the rarity of diseases, research and development of orphan medicines tackle greater complexity because of the limited number of patients to be potentially included in clinical trials, the lack of validated biomarkers, the use of surrogate end-points, and the paucity of available knowledge and medical expertise [2]. Furthermore, the marketing of orphan medicines is expected to have limited financial returns due to high development costs and small market size [3], discouraging companies from investing in products for rare diseases.

To encourage the implementation of medicines for patients affected by rare diseases, the European Parliament and Council adopted the Orphan Medicinal Product Regulation in 2000 [4], establishing terms and condition for orphan designation in the EU and providing incentives for the development and marketing of designated orphan medicines. To apply for orphan designation, a product under development must meet a number of criteria: (i) it must be intended for the prevention, diagnosis or treatment of a life-threatening or chronically debilitating disease; (ii) the prevalence of the disease must not be more than 5 in 10,000 people in the EU, or it must be unlikely, in the absence of incentives, that the marketing of the product would generate sufficient return on investment; (iii) no valid alternative should be available, or, if such alternative exists, the product must be of significant benefit to those affected by the disease [4]. Following a positive opinion from the European Medicines Agency (EMA) Committee for Orphan Medicinal Products (COMP), orphan designation is granted by the European Commission (EC) with access to a range of incentives, including protocol assistance, fee reductions and market exclusivity. Once development is complete, the designated orphan product is evaluated for Marketing Authorisation (MA) by EMA Committee for Medicinal Products for Human Use (CHMP) centrally in the EU; at the same time, compliance with orphan designation criteria is reviewed by the COMP for maintenance of orphan status once the medicine is placed on the market [5].

As the development of a new medicine is generally a lengthy process, the EU Orphan Medicinal Product Regulation was expected to change the therapeutic landscape for patients suffering from rare diseases gradually [1]. The main objective of this study was to describe the key characteristics of medicines with designated orphan indications authorised in EU during the first 24 years of the Orphan Medicinal Product Regulation until January 17, 2024.

## Materials and Methods

The present study was performed on the dataset of the Community Register of orphan medicinal products for human use as of January 17, 2024 [6]. From the EU register, orphan designation applications submitted to the EMA were classified according to their outcome into active, withdrawn or expired, and refused. As the main objective of this study, active orphan designations that resulted in authorised clinical indications of medicinal products were retrieved and included in the study. Data on medicinal products with designated orphan indications were collected from web-based sources using a specially created database including general information on the medicine, the orphan designation, and the MA, and then analysed using descriptive statistics.

General medicine information was retrieved from the Union Register of medicinal products for human use [7], and comprised brand name, active substance, therapeutic indication, and EMA product number. All medicines were classified according to the Anatomical Therapeutic Chemical (ATC) classification system [8], and, depending on the manufacturing process, divided into biologically or chemical drug products. The number of orphan medicines authorised for paediatric use as well as the number of active orphan designations resulted in paediatric indications were assessed.

For orphan designation information, data were collected from the Community Register of orphan medicinal products for human use [9], and included designated orphan indication, EU orphan designation number, and designation date of issue. The prevalence of the condition in the EU was reported as estimated by the COMP at the time of the review of the designation criteria in the EMA Orphan Maintenance Assessment Report or in the EMA Review of Orphan Medicine Designation at the time of Marketing Authorisation, depending on the document available on the web. For those conditions for which a post-authorisation review of orphan designation criteria at the time of type II variations [10] was available, the latest prevalence estimate was reported. Similarly, the existence of satisfactory alternative methods authorised in the EU for patients affected by the condition at the time of the latest review of orphan designation criteria was reported. If none of the cited documents were available, the prevalence of the condition and the existence of satisfactory alternative methods at the time of orphan designation were given. According to EMA’s recommendations [11], conditions were classified according to the International Classification of Diseases 11th Revision (ICD-11) [12].

Information about MA was collected from the Union Register of medicinal products for human use [7] and from the European Public Assessment Reports (EPARs) within the scope of active orphan designations available on the web, including any variations. With regard to EPAR variations, only those concerning changes in the therapeutic indication, such as addition of a new designated orphan indication or modification of an approved one, and resulting in updates to Sect. 4.1 of the Summary of Product Characteristics (SmPC) were considered. On the other hand, EPARs and any variations relating to therapeutic indications with expired, withdrawn or no orphan designation, or relating to the addition of new pharmaceutical forms, new strengths or new routes of administration were not considered for data collection. MA general information included EU MA number, MA holder, MA date of issue, time from designation date to approval date of the designated orphan indication, type of MA, whether the medicine is subject to additional monitoring, granting of two-year extension of market exclusivity, request for scientific advice (SA) or protocol assistance (PA), similarity with authorised orphan medicines, and new active substance status. With regard to the type of MA, orphan designated medicines were evaluated for having received standard MA, conditional MA, or MA under exceptional circumstances. In the interest of public health, the EMA may grant MA for medicines that address unmet medical needs, such as orphan medicines, through specific regulatory procedures. Conditional MA may be granted on the basis of less comprehensive clinical data than normally required, when the benefit of the medicine’s immediate availability outweighs the risk inherent in the fact that additional data are still required [13]. Once granted, conditional MA can be converted into standard once the MA holder fulfils the specific obligations imposed at the time of granting, and provides the necessary data to confirm the benefit/risk balance of the medicine is positive [13]. In the absence of comprehensive clinical data, MA may also be granted under exceptional circumstances. Unlike conditional MA, the holder will not be able to provide complete data even after authorisation as the indication to be treated is too rare, the current scientific knowledge is insufficient, or the collection of data is unethical [14]. For this reason, MA under exceptional circumstances does not normally lead to a switch into standard [13]. Medicines given conditional approval or authorised under exceptional circumstances are subject to additional monitoring, which means that they are being monitored particularly closely by regulatory authorities [15]. Since other medicine categories are also subject to additional monitoring, orphan medicines with additional monitoring included in this study were evaluated according to the EMA List of medicines under additional monitoring updated in January 2024 [16]. Regarding the incentives provided by the EMA, the number of medicines that received SA or PA in therapeutic indications with active orphan designation, as well as the number of orphan designated indications for which a two-year extension of market exclusivity was granted were assessed. For all medicinal products for human use, SA may be requested from the EMA on any aspect of the various studies and trials relating to the evaluation of quality, safety and efficacy [17]. In addition, designated orphan products may receive a specific type of SA, called PA, concerning quality, non-clinical and clinical aspects of the development of the medicine within the designated orphan indication [17]. Once MA is granted and orphan status is maintained, designated orphan medicines also benefit from 10-year market exclusivity, i.e. the protection from market competition with similar medicines for the same therapeutic indication for a period of 10 years after approval [18]. Two additional years of market exclusivity may be granted for orphan indications with the results of studies addressing the paediatric population in accordance to an agreed paediatric investigation plan included in the SmPC, leading to a total of 12 years of market protection [18]. Finally, within the scope of this study, the number of orphan medicines for which a similarity report was submitted, and which qualified as new active substances was assessed. For all medicinal products, regardless of orphan designation, a critical report addressing the possible similarity with orphan medicines under market exclusivity protection authorised for the indication claimed for the new medicinal product must be submitted at the time of application for MA and any post-authorisation variations. If there is no authorised orphan medicine for a condition related to the proposed indication, no similarity report will be required for approval [19]. With regard to new active substance status, the CHMP, based on the available data, may qualify the active substance contained in the medicinal product applying for MA as new if consistent with the definition of new chemical, biological or radiopharmaceutical active substance given by the EC [20].

## Results

**Fig. 1:**
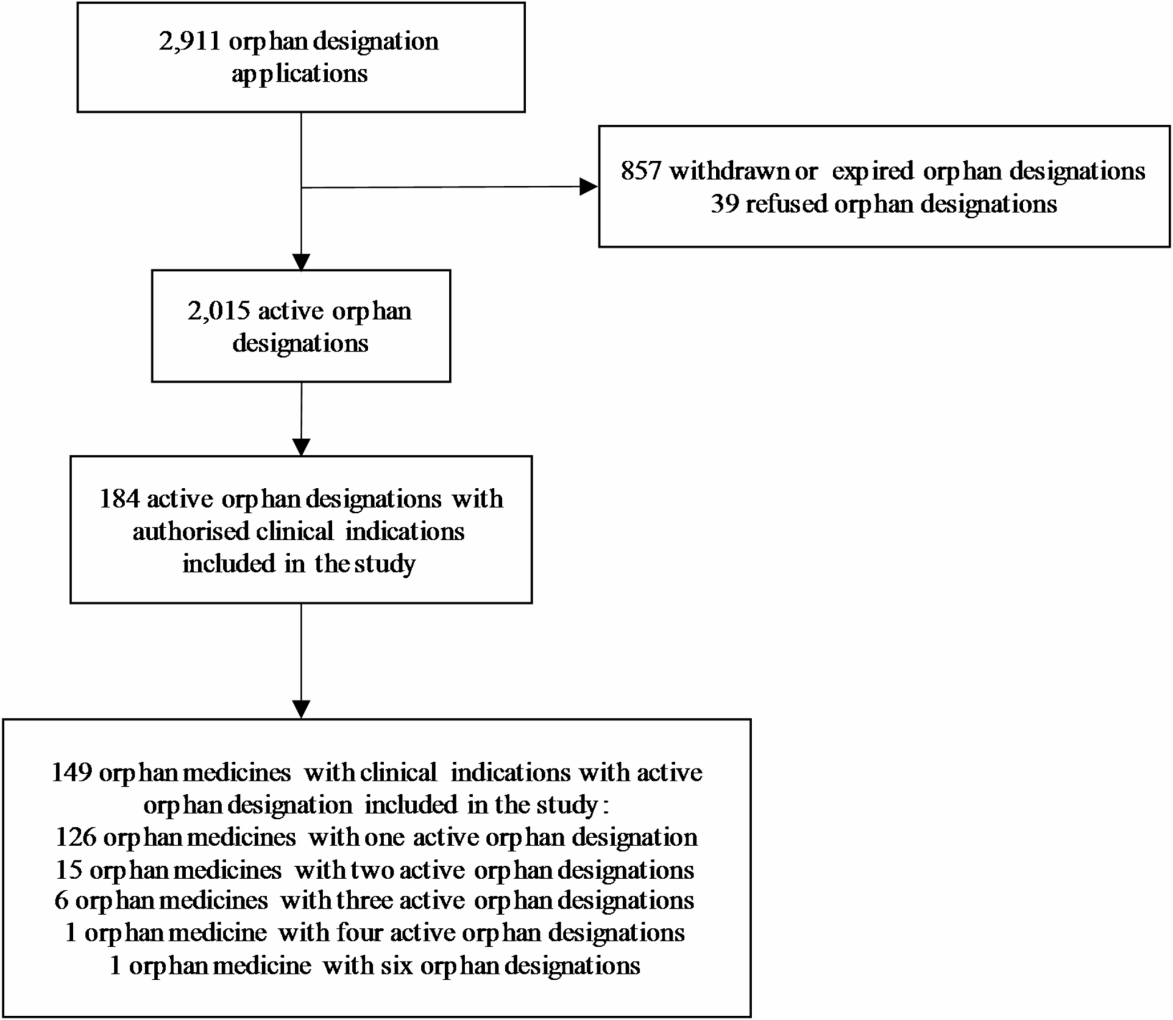
Flow diagram of the selection of included orphan designations and included orphan medicines

From the entry into force of the Orphan Medicinal Product Regulation in 2000 until 17 January 2024, a total of 2,911 orphan designation applications have been submitted to the EMA, including 2,015 active orphan designations (69.2%), 857 withdrawn or expired orphan designations (29.4%), and 39 refused orphan designations (1.4%). Of the 2,015 active orphan designations, 184 resulted in authorised clinical indications (6.3%), making a total of 149 orphan medicinal products included in the study (Fig. 1). Covering multiple therapeutic indications for rare diseases, 23 (15.4%) orphan medicines accounted for several distinct active orphan designations, resulting in the number of designations exceeding the number of medicines (Fig. 1).

**Fig. 2:**
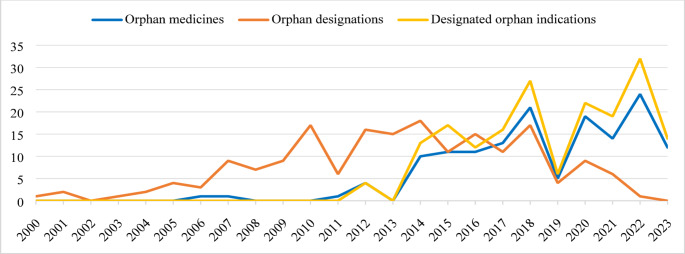
Number of orphan medicines, orphan designations and orphan designated indications included during the study period

As shown in Fig. 2, the number of orphan medicines has progressively increased during the study period, with peaks recorded in 2018 and 2022 and with the largest number of medicines authorised between 2020 and 2024 (Table 1). According to the ATC classification, antineoplastic and immunomodulatory agents accounted for the highest number of orphan medicines (58; 38.9%), with almost one third being antineoplastic agents (47; 31.5%) (Table 1). By type of product, 65 (43.6%) orphan medicines were biological products, mainly monoclonal antibodies (18; 12.1%), recombinant human peptides or enzymes (14; 9.4%), or gene therapies (12; 8.1%) (Table 1). Based on the CHMP review, 123 (82.6%) active substances were qualified as new as they were not constituents of a medicinal product previously authorised within the EU (Table 1). Specifically, 95.4% of biological products were considered new substances, compared to 72.6% of chemical drug products. Of the total 149 authorised orphan medicines, most (96; 64.4%) received standard MA, 33 (22.1%) conditional MA, and 20 (13.4%) MA under exceptional circumstances (Table 1). Among the conditional MAs granted, 10 (6.7%) switched to non-conditional after a mean of 50.7 months (range 11–114 months). The number of medicines for which at least one SA or PA was requested for the approval of a clinical indication with active orphan designation has increased significantly over the years, reaching 94.4% of newly authorised orphan products in the period 2000–2024 (Table 1). Regarding the MA application of Palynziq^®^ for the treatment of phenylketonuria, the applicant did not seek SA or PA in the EU. However, the same applicant obtained SA for the previous approval of Kuvan^®^, also for the treatment of patients with phenylketonuria, in which blood phenylalanine levels were accepted as a surrogate endpoint. The same endpoint was also used during the clinical development of Palynziq^®^ [21].

**Table 1:** General characteristics of orphan medicines included in the study

	Orphan medicines with MA granted 2005–2009 (*n* = 2)		Orphan medicines with MA granted 2010–2014 (*n* = 15)	Orphan medicines with MA granted 2015–2019 (*n* = 61)	Orphan medicines with MA granted 2020–2024^a^ (*n* = 71)	Orphan medicines with MA granted 2005–2024^a^ (*n* = 149)
**ATC 1st level**, ***n*** **(%)**^**b**^						
A – Alimentary tract and metabolism			2 (1.3)	15 (10.1)	12 (8.1)	29 (19.5)
B – Blood and blood forming organs				5 (3.4)	6 (4.0)	11 (7.4)
C – Cardiovascular system				2 (1.3)		2 (1.3)
D – Dermatologicals			1 (0.7)		1 (0.7)	2 (1.3)
H – Systemic hormonal preparations, excl. Sex hormones and insulins			2 (1.3)	1 (0.7)	7 (4.7)	10 (6.7)
J – Antiinfectives for systemic use			3 (2.0)	2 (1.3)	6 (4.0)	11 (7.4)
L – Antineoplastic and immunomodulating agents	2 (1.3)		5 (3.4)	20 (13.4)	31 (20.8)	58 (38.9)
M – Musculo-skeletal system			1 (0.7)	2 (1.3)	2 (1.3)	5 (3.4)
N – Nervous system			1 (0.7)	6 (4.0)	3 (2.0)	10 (6.7)
P – Antiparasitic products, insecticides and repellents					1 (0.7)	1 (0.7)
R – Respiratory system				1 (0.7)	1 (0.7)	2 (1.3)
S – Sensory organs				5 (3.4)		5 (3.4)
V – Various				2 (1.3)	1 (0.7)	3 (2.0)
**Product type**, ***n*** **(%)**^**b**^						
Chemically derived product	1 (0.7)		10 (6.7)	34 (22.8)	39 (26.2)	84 (56.4)
Biological products	1 (0.7)		5 (3.4)	27 (18.1)	32 (21.5)	65 (43.6)
Antibody-drug conjugate			1 (0.7)	2 (1.3)	2 (1.3)	5 (3.4)
Cell therapy				2 (1.3)	1 (0.7)	3 (2.0)
Gene therapy				4 (2.7)	8 (5.4)	12 (8.1)
Human antibody fragment					1 (0.7)	1 (0.7)
Human coagulation factor X				1 (0.7)		1 (0.7)
Monoclonal antibody	1 (0.7)		2 (1.3)	6 (4.0)	9 (6.0)	18 (12.1)
Nanobody				1 (0.7)		1 (0.7)
Recombinant bacterial enzyme				1 (0.7)	2 (1.3)	3 (2.0)
Recombinant fusion protein				3 (2.0)	4 (2.7)	7 (4.7)
Recombinant human enzyme/peptide			2 (1.3)	7 (4.7)	5 (3.4)	14 (9.4)
**New active substance**, ***n*** **(%)**^**b**^						
Yes	2 (1.3)		13 (8.7)	46 (30.9)	62 (41.6)	123 (82.6)
No			2 (1.3)	15 (10.1)	9 (6.0)	26 (17.4)
**Type of initial MA**, ***n*** **(%)**^**b**^						
Standard MA	2 (1.3)		8 (5.4)	45 (30.2)	41 (27.5)	96 (64.4)
Conditional MA			5 (3.4)	8 (5.4)	20 (13.4)	33 (22.1)
MA under exceptional circumstances			2 (1.3)	8 (5.4)	10 (6.7)	20 (13.4)
**Additional monitoring**, ***n*** **(%)**^**b**^						
Yes			9 (6.0)	22 (14.8)	64 (43.0)	95 (63.8)
No	2 (1.3)		6 (4.0)	39 (26.2)	7 (4.7)	54 (36.2)
**SA/PA requested**, ***n*** **(%)**^**b**^						
Yes	1 (0.7)		11 (7.4)	50 (33.6)	67 (45.0)	129 (86.6)
No	1 (0.7)		4 (2.7)	11 (7.4)	4 (2.7)	20 (13.4)

^a^ Until January 17, 2024.

^b^ The percentages refer to the total number of orphan medicines included in the study.

The 184 active orphan designations with authorised clinical indications covered a total of 135 different main indications, of which almost 98% were for the treatment of rare diseases while the rest was for prevention or diagnosis of rare diseases (Table 2). According to the ICD-11 classification, most of the orphan designations concerned indications for neoplasms (60; 32.6%), or endocrine, nutritional or metabolic diseases (49; 26.6%) (Table 2). In the area of oncology, most were for the treatment of neoplasms of haematopoietic or lymphoid tissues (44; 23.9%), including 7 for the treatment of acute myeloid leukaemia, 7 for the treatment of multiple myeloma, 6 for the treatment of diffuse large B-cell lymphoma, 4 for the treatment of follicular lymphoma, 3 for the treatment of cutaneous T-cell lymphoma, 2 for the treatment of acute lymphoblastic leukaemia, and 2 for the treatment of mastocytosis. As for indications for endocrine, nutritional or metabolic diseases with multiple orphan designations, the treatment of growth hormone deficiency and the treatment of transthyretin-mediated amyloidosis counted 3 designations respectively, while the treatment of acromegaly, Cushing’s syndrome, hyperargininaemia, hypoparathyroidism counted 2 designations each. Since 2000, the number of active orphan designations granted has increased gradually until 2014, before decreasing sharply over the last years (Fig. 2). The number of designated orphan indications, on the other hand, has progressively increased during study period, following a similar trend as that for authorised orphan medicines (Fig. 2). Designated orphan indications were licensed after a mean of 67.2 months (range 6–249 months) from designation date (Table 2), with a total of 17 (9.2%) approved post-authorisation as addition of a new therapeutic indication to an existing MA. Withdrawn from the Community Register of designated orphan medicinal products at the time of MA, Trecondi^®^ was initially authorised as a non-orphan medicinal product in June 2019. However, further to the judgment of the General Court, the EC adopted an implementing decision on 24 November 2020, authorising the medicine as orphan [22]. Exceptionally, the time from designation date to the approval date of the orphan indication claimed by Trecondi^®^ was assessed considering the November 24, 2020, as the approval date. Of the 184 active orphan designations, 93 (50.5%) were for conditions with a prevalence estimate ≤ 1/10,000, including 39 (21.2%) for conditions affecting less than 1 in 50,000 people in the EU (Table 2). Among orphan medicines for extremely rare conditions, Nyxthracis^®^ has been authorised for the treatment of anthrax which accounted for the condition with the lowest prevalence estimate of less than 0.001 per 10,000, followed by Mepsevii^®^ for the treatment of Sly syndrome and Myalepta^®^ for the treatment of Lawrence syndrome for which the conditions were estimated to affect 0.001 in 10,000 European citizens. For a total of 11 (6.0%) designated orphan indications, criteria for orphan designation were reviewed by the COMP post-authorisation. For four indications the prevalence of the condition was estimated to be higher than at the time of MA, for two the prevalence estimate was lower, and for five unchanged. With regard to Luxturna^®^, orphan designation was granted for two different indications, the treatment of Leber’s congenital amaurosis in April 2012 and the treatment of retinitis pigmentosa in July 2015. However, the applicant was invited to amend the prevalence to account for all inherited retinal dystrophies for orphan maintenance report, which was estimated at approximately 3 out of 10,000. Since they were granted in different years, the two designations have been considered separately in Table 2, each with a prevalence estimate of about 3. For a total of 59 (32.1%) orphan indications, no satisfactory methods were authorised in the EU for patients affected by the condition at the time of the latest review of orphan designation criteria (Table 2). For Oxbryta^®^, Nulibry^®^, and Hyftor^®^, the review of the orphan designation at the time of MA and any variations was not available, so the estimated prevalence of the condition and the existence of satisfactory alternative methods were reported as at the time of orphan designation. Similarity to authorised orphan medicines (i.e. a medicinal product containing a similar active substance or substances as contained in a currently authorised orphan medicinal product, and which is intended for the same therapeutic indication) for which a period of market exclusivity was in force was addressed for 75 (40.8%) designated orphan indications claimed by a total of 69 (46.3%) medicines included in the study (Table 2). With regard to market exclusivity, two additional years were granted as paediatric reward for a total of 39 (21.2%) indications with orphan designation (Table 2), making a total of 32 (21.5%) medicines with 12 years of market protection in at least one orphan indication. Nevertheless, 84 (45.7%) orphan designations resulted in paediatric indications (Table 2), with a total of 69 (46.3%) medicines authorised for paediatric use. Specifically, of the 39 (21.2%) designated orphan indications with a two-year extension of market exclusivity, 35 included paediatric indications.

**Table 2:** General characteristics of orphan designations included in the study

	Orphan designations granted 2000–2004 (*n* = 6)		Orphan designations granted 2005–2009 (*n* = 32)	Orphan designations granted 2010–2014 (*n* = 72)	Orphan designations granted 2015–2019 (*n* = 58)	Orphan designations granted 2020–2024^a^ (*n* = 16)	Orphan designations granted 2000–2024^a^ (*n* = 184)
**Indication for rare diseases**, ***n*** **(%)**^**b**^							
Treatment	6 (3.3)		31 (16.8)	71 (38.6)	56 (30.4)	16 (8.7)	180 (97.8)
Prevention			1 (0.5)	1 (0.5)	1 (0.5)		3 (1.6)
Diagnosis					1 (0.5)		1 (0.5)
**ICD-11 chapter**, ***n*** **(%)**^**b**^							
01 Certain infectious or parasitic diseases			4 (2.2)	6 (3.3)	2 (1.1)	2 (1.1)	14 (7.6)
02 Neoplasms	2 (1.1)		7 (3.8)	18 (9.8)	22 (12.0)	11 (6.0)	60 (32.6)
03 Diseases of the blood or blood-forming organs			3 (1.6)	3 (1.6)	5 (2.7)		11 (6.0)
04 Diseases of the immune system			2 (1.1)	2 (1.1)	1 (0.5)		5 (2.7)
05 Endocrine, nutritional or metabolic diseases	1 (0.5)		10 (5.4)	25 (13.6)	11 (6.0)	2 (1.1)	49 (26.6)
07 Sleep-wake disorders			1 (0.5)	1 (0.5)			2 (1.1)
08 Diseases of the nervous system			2 (1.1)	6 (3.3)	7 (3.8)	1 (0.5)	16 (8.7)
09 Diseases of the visual system			2 (1.1)	1 (0.5)	2 (1.1)		5 (2.7)
11 Diseases of the circulatory system				2 (1.1)			2 (1.1)
12 Diseases of the respiratory system					2 (1.1)		2 (1.1)
13 Diseases of the digestive system	1 (0.5)		1 (0.5)	2 (1.1)			4 (2.2)
14 Diseases of the skin				2 (1.1)			2 (1.1)
15 Diseases of the musculoskeletal system or connective tissue				1 (0.5)			1 (0.5)
16 Diseases of the genitourinary system					1 (0.5)		1 (0.5)
19 Certain conditions originating in the perinatal period					1 (0.5)		1 (0.5)
20 Developmental anomalies				3 (1.6)	3 (1.6)		6 (3.3)
23 External causes of morbidity or mortality	1 (0.5)						1 (0.5)
24 Factors influencing health status or contact with health services	1 (0.5)				1 (0.5)		2 (1.1)
**Δ years**, ***n*** **(%)**^**b**^							
Δ ≤ 3				10 (5.4)	23 (12.5)	15 (8.2)	48 (26.1)
3 < Δ ≤ 6			6 (3.3)	34 (18.5)	28 (15.2)	1 (0.5)	69 (37.5)
6 < Δ ≤ 9			16 (8.7)	19 (10.3)	7 (3.8)		42 (22.8)
9 < Δ ≤ 12	1 (0.5)		5 (2.7)	9 (4.9)			15 (8.2)
12 < Δ ≤ 15	1 (0.5)		5 (2.7)				6 (3.3)
15 < Δ ≤ 18	2 (1.1)						2 (1.1)
18 < Δ ≤ 21	2 (1.1)						2 (1.1)
**Prevalence per 10**,**000**, ***n*** **(%)**^**b**^							
≤ 1	5 (2.7)		17 (9.2)	41 (22.3)	27 (14.7)	3 (1.6)	93 (50.5)
≤ 2	5 (2.7)		23 (12.5)	53 (28.8)	37 (20.1)	7 (3.8)	125 (67.9)
≤ 3	6 (3.3)		25 (13.6)	60 (32.6)	44 (23.9)	10 (5.4)	145 (78.8)
≤ 4	6 (3.3)		30 (16.3)	68 (37.0)	50 (27.2)	10 (5.4)	164 (89.1)
≤ 5	6 (3.3)		32 (17.4)	72 (39.1)	58 (31.5)	16 (8.7)	184 (100)
**Existence of satisfactory alternative methods**, ***n*** **(%)**^**b**^							
Yes	4 (2.2)		22 (12.0)	47 (25.5)	38 (20.7)	14 (7.6)	125 (67.9)
No	2 (1.1)		10 (5.4)	25 (13.6)	20 (10.9)	2 (1.1)	59 (32.1)
**Similarity to authorised orphan medicines**, ***n*** **(%)**^**b**^							
Yes	3 (1.6)		9 (4.9)	24 (13.0)	28 (15.2)	11 (6.0)	75 (40.8)
No	3 (1.6)		23 (12.5)	48 (26.1)	30 (16.3)	5 (2.7)	109 (59.2)
**Two-year extension of market exclusivity**, ***n*** **(%)**^**b**^							
Yes	3 (1.6)		13 (7.1)	16 (8.7)	4 (2.2)	3 (1.6)	39 (21.2)
No	3 (1.6)		19 (10.3)	56 (30.4)	54 (29.3)	13 (7.1)	145 (78.8)
**Paediatric indication**, ***n*** **(%)**^**b**^							
Yes	5 (2.7)		17 (9.2)	33 (17.9)	26 (14.1)	3 (1.6)	84 (45.7)
No	1 (0.5)		15 (8.2)	39 (21.2)	32 (17.4)	13 (7.1)	100 (54.3)

^a^ Until January 17, 2024.

^b^ The percentages refer to the total number of orphan designations included in the study.

## Discussion

The main objective of this study was to describe the regulatory key characteristics of medicines with designated orphan indications authorised in EU during the first 24 years of the Orphan Medicinal Product Regulation. Although previous studies have provided valuable insights into the European experience with orphan products [23–26], this study included the most recent advances in the field of rare diseases, providing a current overview of the availability of orphan medicines in the EU.

By the beginning of 2024, 149 medicines with clinical indications with active orphan designation had been authorised by the EC, with a total of 184 active orphan designations resulting in a positive opinion for MA. Prior to the introduction of the Orphan Medicinal Product Regulation in 2000, treatment options for patients suffering from rare diseases were either limited or non-existent, setting the stage for a specific legislative framework in this area of huge unmet need [1]. Given the gradual increase in both the number of orphan medicines and orphan designated indications in the last few years, the EU Orphan Medicinal Product Regulation has confirmed undeniable beneficial effects in fostering the development and marketing of orphan products, as highlighted by the latest evaluation of the EC on the period 2000–2017 [1]. The constant dialogue between researchers, clinicians and regulatory authorities, which are organised through networks of centres of expertise and healthcare, has certainly contributed to this phenomenon, allowing for the acceleration of the identification of new therapies also thanks to multinational clinical trials.

Patient associations also play an important role, having a priority role in the coordination and dissemination of data, both for the search for specialised centres and participation in clinical trials. Even some non-profit foundations are making a significant contribution to the marketing of orphan drugs in EU: this is the case of the Italian research charity Telethon Foundation which has decided to acquire the license for the production and distribution of Strimvelis^®^, a gene therapy product that represents the only available cure for a rare disease called ADA-SCID. Furthermore, the same foundation announced that it has submitted the Marketing Authorization Application (MAA) for the gene therapy - etuvetidigene autotemcel - for the treatment of patients with Wiskott-Aldrich Syndrome (WAS), a rare genetic disease of the immune system, to the EMA [25]. Despite the increasing availability of medicines for rare diseases, still a small proportion of designated orphan products have been developed and reached the market. Indeed, of the 2,015 active orphan designations granted to date, 9.1% have been actually translated into authorised clinical indications, highlighting the discrepancy between the number of orphan designations and MAs and indicating that there is still much work to be done to give patients with rare diseases hope of a cure. Among the possible reasons to justify the limited number of orphan drugs we could hypothesize the uncertainty of the diagnosis, that is the difficulty in identifying the pathology, the complexity of the pathology itself and the low frequency with which it appears which results in a small number of patients (with subsequent recruitment issues) but also adaptive designs failing and though post-marketing commitments.

Of the184 orphan designations with approved clinical indications in the EU, nearly one third were in the oncology area, most being for the treatment of neoplasms of haematopoietic or lymphoid tissues. The clustering of orphan medicines in oncology was previously evidenced in the EC evaluation [1], and, according to this study, further 31 out of 97 (32.0%) newly authorised orphan products gained MA as antineoplastic agents (i.e. ATC code L01) since 2018. Although medicines targeting rare diseases are expected to have low financial rewards, the high proportion of antineoplastic orphan products may suggest oncology is a more profitable therapeutic area for the pharmaceutical industry due to the broader applicability of medicines to different types of cancer [1]. Given the growing number of orphan medicines in oncology and around 32% of orphan indications authorised for conditions with no satisfactory alternatives, regulatory interventions in less economically attractive areas for which no treatments exist are required to address persistent market failures in rare diseases. However, although oncological designations are still very important, in the last five years these have been markedly reduced. This drop in designations is not particularly surprising given the high number of designations in previous years and the number of drugs actually marketed. As a consequence, companies may have decided to shift their investments from oncology drugs to new therapies (such as gene therapies, therapeutic RNA vaccines, CAR-T not only in the oncohematological field, CRISPR therapies, etc.).

A further unmet medical need is that of rare childhood diseases, which remain a neglected area for the development and marketing of medicines. Considering that the majority of rare diseases affect children and that less than half of the orphan indications were authorised in certain paediatric subgroups, regulatory efforts have been implemented in paediatrics. The further 2 years extension of the Marketing Exclusivity has not been as successful as expect with only around 40 being granted since the start of the paediatric legislation after 2006. The difficulties in getting a Paediatric Investigational Plan then completing it meeting compliance criteria is tough, together with the problems of doing small patient population trials in paediatric populations.

Regarding the type of MA, most of the orphan products in this study entered the market with standard MA, with only 22.1% and 13.4% receiving conditional MA and MA under exceptional circumstances, respectively. Assessing the methodological quality of orphan medicinal product dossiers, the previous study by Joppi et al. reported that efficacy and safety profiles of medicines for rare diseases were worryingly lacking [26]. Despite small improvements in development methods over time, the number of patients studied, the use of placebos and surrogate endpoints, and the follow up period have been often inadequate [26]. Considering the less comprehensive clinical data resulting from these methodological issues, orphan products addressing unmet medical needs were expected to gain MA through specific regulatory procedures, which is contrast to the more than 60% of standard MA granted in this study. However, it is worth noting that conditional MA issued in recent years have increased sharply, almost tripling in the period 2020–2024 compared to the period 2015–2019, which suggests greater stringency in the evaluation of MA applications in the last few years.

Among the various incentives provided by the EMA to encourage the marketing of medicines for rare diseases, SA and PA enable developers of designated orphan products to receive dedicated development support in the pre-marketing phase, improving compliance with EU regulatory standards. According to this study, the number of medicines seeking SA or PA within the scope of orphan indications has increased significantly over the years, and approximately 94% of the orphan medicines authorised from 2020 to 2024 have benefited from it. Among determinants of MA applications, compliance with SA was found to be statistically significantly associated with successful MA applications of orphan products [27]. As reported in a previous retrospective study [27], applications with not acceptable clinical development plan during SA assessment were 76% more likely to receive a positive opinion from the CHMP when the advice to change the plan to comply with recommendations was followed, showing positive effects of SA on the development program and, ultimately, on the MA application success. Although compliance with the SA or PA recommendations was not assessed in this study, the significant increase in the number of requests in recent years may suggest that developers of orphan products have become more aware of the benefits of EU incentives on MA application outcome, as called for in the study by Hofer et al. [27].

It is worth noting that the availability of orphan medicinal products authorised by the EC does not translate into equal accessibility in all Member States. Despite MA being granted centrally in the EU, access to authorised orphan medicines may vary considerably between European countries, mainly because of different national pricing policies, reimbursement systems, and companies’ strategic launch decisions [1]. To try to overcome these national limits, a group named AGORA (Access to Gene Therapies for Rare Disease, ) was founded in 2022 that brings together stakeholders from across the UK and EU who represent academic groups, regulators, funders, patient advocacy groups and drug developers with the aims to create a sustainable solution for children with rare and ultra-rare diseases to access novel gene therapies that are shown to be effective in clinical trials [28].The present study should be considered in the light of the following limitations. The purpose of this descriptive analysis was to provide a current overview of the availability of orphan medicines in EU by retrieving products with active orphan designation that have been granted MA by the EC. However, medicines may be approved for one or more clinical indications of rare diseases without having gained orphan designation, and medicines that has been granted MA as orphan may now have their orphan designation expired or withdrawn. In order to provide a more comprehensive overview of medicines for patients suffering from rare diseases, all medicines, orphan or otherwise, authorised in the EU for rare diseases should be evaluated. Finally, the present study was conducted on general information on orphan medicines, orphan designations, and MAs collected from web-based sources. Despite being readily and publicly available, information obtained from the web may not be up-to-date, or, on the other hand, may not be available at all, as in the case of some reviews of orphan designation criteria at the time of MA. Besides potentially less accurate data, the orphan drug landscape in the EU is continuously changing, with orphan designations progressively expiring or being removed from Community Register of orphan medicinal products for human use, or orphan medicines even being withdrawn from the market. Nevertheless, this study has provided a current overview of the actual availability of orphan medicines in the EU, showing pharmacological advances since the Orphan Medicinal Product Regulation came into force in 2000.

## Conclusions

Despite the increase of orphan products placed on the market over the years, the number of authorised orphan indications is still low compared to the total number of orphan designations granted and to the more than six thousand rare diseases estimated, making the lack of available medicines for therapeutic areas such as childhood rare diseases and rare diseases with no available treatments a priority for regulatory interventions.

## Data Availability

The data that support the findings of this study are available from the corresponding author upon reasonable request.
